# Racial, socioeconomic, and payer status disparities in utilization of unicompartmental knee arthroplasty in the USA

**DOI:** 10.1186/s43019-024-00227-4

**Published:** 2025-01-09

**Authors:** Suraj A. Dhanjani, Jessica Schmerler, Nauman Hussain, Daniel Badin, Uma Srikumaran, Vishal Hegde, Julius K. Oni

**Affiliations:** 1 Department of Orthopedic Surgery, Mount Sinai Health System, 5 East 98 Street, New York, NY 10029 USA; 2 Department of Orthopedic Surgery, The Johns Hopkins University School of Medicine, 601 N Caroline St., Baltimore, MD 21287 USA; 3 Department of Orthopaedic Surgery, The Johns Hopkins University, 4940 Eastern Avenue, Bayview Medical Offices Building, 1st Floor, Baltimore, MD 21224 USA

**Keywords:** Unicompartmental knee arthroplasty, Total knee arthroplasty, Racial disparities, Socioeconomic disparities, Payer status disparities

## Abstract

**Background:**

Unicompartmental knee arthroplasty (UKA) is a surgical treatment for knee osteoarthritis associated with lower morbidity compared with total knee arthroplasty (TKA) in patients with isolated unicompartmental knee arthritis. As disparities have been noted broadly in arthroplasty care, it follows that such disparities might be present in the utilization of UKA relative to TKA. This study therefore examined racial/ethnic, socioeconomic, and payer status differences in utilization of UKA.

**Methods:**

Patients who underwent UKA or TKA between 2016 and 2020 in the National Inpatient Sample were identified. Multivariable Poisson regression models adjusted for hospital geographic region and patient characteristics [age, sex, and Elixhauser Comorbidity Index (ECI)] were used to examine the effect of race/ethnicity, socioeconomic status, and payer status on incidence rate ratio of UKA relative to TKA.

**Results:**

Of the 8472 UKA patients and 639,937 TKA patients identified between 2016 and 2020, 8027 (94.7%) UKA patients and 606,028 (94.7%) TKA patients met inclusion criteria. Patients who underwent UKA were significantly younger (63.5 ± 10.7 years) than patients who underwent TKA (66.8 ± 9.5 years; p < 0.001) and had significantly lower ECI scores (1.8 ± 1.5) than patients who underwent TKA (2.2 ± 1.6; *p* < 0.001). Black patients were less likely to undergo UKA relative to TKA compared with white patients [incidence rate ratio (IRR) 0.64, confidence interval (CI) 0.58–0.71, *p* < 0.001]. Compared with patients in income quartile 4, patients in income quartiles 1 and 2 underwent UKA at a lower relative rate (IRR 0.85, CI 0.79–0.90, *p* < 0.001 and IRR 0.87, CI 0.82–0.93, *p* < 0.001, respectively). Compared with patients with private insurance, patients with Medicare underwent UKA at a lower relative rate (IRR 0.83, CI 0.79–0.88, *p* < 0.001).

**Conclusions:**

Black patients, lower-income patients, and Medicare-insured patients undergo UKA at a lower relative rate than white, higher-income, and privately insured patients, respectively. Further research may help elucidate reasons for these differences and identify targets for intervention.

**Supplementary Information:**

The online version contains supplementary material available at 10.1186/s43019-024-00227-4.

## Background

Osteoarthritis (OA) of the knee is one of the most common ailments in the US population, affecting 9.3 million adults over the age of 45 years old [1]. The burden of this disease is quickly growing as the population continues to age. This is evidenced by the fact that utilization of total knee arthroplasty (TKA), the most common form of surgical management, has exponentially increased over the last decade [2, 3]. Unicompartmental knee arthroplasty (UKA) is another increasingly utilized option for surgical treatment of knee osteoarthritis shown to be associated with lower morbidity, faster recovery, and greater patient satisfaction compared with TKA in patients with isolated unicompartmental knee osteoarthritis who meet other indications for UKA, such as minimal to no flexion contracture, minimal varus/valgus deformity, and ligamentous stability [4–7]. Furthermore, although UKA has in some studies been shown to be associated with a higher revision rate than TKA and conversion to TKA, several studies have shown that UKA is a cost efficient treatment for knee osteoarthritis in the USA and abroad [8–10]. Because of these advantages, it is important to ensure equitable access to UKA. Additionally, as UKA currently comprises only about 4% of knee arthroplasties performed in the USA [11], it is critical that any future growth in utilization of the procedure does not perpetuate any inequities in access.

Previous studies have shown differences in utilization of TKA based on race. Specifically, Atarere et al. showed that Black and Hispanic patients have been 13% and 11% less likely to undergo TKA compared with white patients, respectively [12]. Socioeconomic factors may also play a role, as patients with lower net worth have been shown to be less likely to undergo TKA compared with patients with higher net worth [13]. Similarly, patients with Medicaid are less likely to obtain knee arthroplasty clinic appointments and later receive TKA compared with patients with private insurance [12, 14]. While these metrics have been well studied in TKA, UKA is an area that has been underexplored. Previous literature that has examined UKA has only done so in the context of race, finding that minority patients, especially Black individuals, were less likely to receive UKA for treatment of isolated knee osteoarthritis [15–17]. One study found a lower rate of UKA in patients of lower socioeconomic status, as defined by Medicare buy-in, but this was not the primary outcome of the study and was not analyzed statistically or using any kind of multivariable analysis [17]. Thus, rare studies have examined disparities in utilization based on socioeconomic status or insurance status, both important components of social determinants of health.

This study seeks to fill the gap in the literature by aiming to answer the following question using a nationally representative dataset: Is there a difference in the utilization of UKA relative to TKA between racial/ethnic, socioeconomic, and differentially insured patient groups? We hypothesized that minority patients, patients with lower socioeconomic status, and publicly insured patients would utilize UKA at a lower rate relative to TKA.

## Methods

This study was exempt from institutional review board approval.

### Data source and study population

This Level III retrospective cohort study used the National Inpatient Sample (NIS), maintained by the Healthcare Cost and Utilization Project (HCUP). The NIS is the largest, publicly available all-payer inpatient database that includes data on > 125 clinical variables, containing data on more than 7 million hospital stays [18]. We included patients over the age of 18 years who underwent either a TKA or a UKA in 2016 through 2020, identified using International Classification of Diseases, Tenth Revision (ICD-10) procedure codes (Supplemental Table 1). We excluded any patients with missing data on the variables or covariates analyzed.

**Table 1:** Characteristics of patients undergoing UKA and TKA

Parameter	Total sample	UKA	TKA	*p*-Value^*^
N	614,055	8027 (1.3%)	606,028 (98.7%)	< 0.001
Age (SD)	66.7 (9.5)	63.5 (10.7)	66.8 (9.5)	
ECI (SD)	2.23 (1.6)	1.80 (1.5)	2.24 (1.6)	
Sex (%)				
Men	237,502 (38.7%)	3916 (48.8%)	233586 (38.5%)	< 0.001
Women	376,553 (61.3%)	4111 (51.2%)	372,442 (61.5%)	
Race/ethnicity (%)				
White	498,133 (81.1%)	6724 (83.8%)	491,409 (81.1%)	< 0.001
Black	51,798 (8.4%)	435 (5.4%)	51,363 (8.5%)	
Hispanic	38,354 (6.3%)	507 (6.3%)	37,847 (6.3%)	
Asian or Pacific Islander	9393 (1.5%)	130 (1.4%)	9263 (1.5%)	
Native American	2761 (0.5%)	38 (0.5%)	2723 (0.5%)	
Other	13,626 (2.2%)	193 (2.4%)	13,423 (2.2%)	
Socioeconomic status (%)				
Income Q1	138,288 (22.5%)	1518 (18.9%)	136,770 (22.6%)	< 0.001
Income Q2	163,390 (26.6%)	1999 (24.9%)	161,391 (26.6%)	
Income Q3	164,877 (26.9%)	2280 (28.4%)	162,597 (26.8%)	
Income Q4	147,500 (24.0%)	2230 (27.8%)	145,270 (24.0%)	
Payer status (%)				
Medicare	352,277 (57.4%)	3409 (42.5%)	348,868 (57.6%)	< 0.001
Medicaid	27,173 (4.4%)	430 (5.4%)	26,743 (4.4%)	
Private	210,705 (34.3%)	3733 (46.5%)	206,972 (34.2%)	
Self-pay	3023 (0.5%)	46 (0.6%)	2977 (0.5%)	
No charge	259 (0.04%)	^**^	254 (0.04%)	
Other	20,618 (3.4%)	404 (5.0%)	20,214 (3.3%)	
Hospital region (%)				
Northeast	119,527 (19.5%)	1858 (23.2%)	117,669 (19.4%)	< 0.001
Midwest	154,157 (25.1%)	1971 (24.6%)	152,186 (25.1%)	
South	226,464 (36.9%)	2490 (31.0%)	223,974 (37.0%)	
West	113,907 (18.6%)	1708 (21.3%)	112,199 (18.5%)	

Q1, quartile 1; Q2, quartile 2; Q3, quartile 3; Q4, quartile 4;SD, standard deviation

^*^p-Values represent chi-squared values, with the exception of age and ECI, which represent *t*-test values

^**^Cells with values ≤ 10 and their corresponding rows are not reported to avoid deidentification of NIS data

Of the 8472 UKA patients and 639,937 TKA patients over the age of 18 years identified, 445 (5.3%) UKA patients and 33,909 (5.3%) TKA patients were excluded due to missing data. Among those with data on race/ethnicity (96.5% of UKA patients and 96.2% of TKA patients), 1.5% of white patients in the initial population, 1.8% of Black patients, 1.8% of Hispanic patients, 1.1% of Asian or Pacific Islander patients, 7.7% of Native American patients, and 1.7% of patients of other races were excluded for missing data on other covariates.

### Variables of interest

*Outcomes of interest* Our primary outcome was incidence rate of UKA relative to TKA (UKA:TKA) by race/ethnicity, socioeconomic status, and payer status. Races/ethnicities analyzed were White, Black, Hispanic, Asian or Pacific Islander (API), Native American, and other. Socioeconomic status was defined using the proxy of quartile (Q) classification (income quartiles Q1–Q4) of the estimated median household income of residents in the patient’s zip code, as laid out in the NIS Description of Data Elements, which states income information is obtained from Claritas Inc. **(**Supplemental Table 2**)**. Payer statuses analyzed were Medicare, Medicaid, private insurance, or self-pay.

**Table 2:** Incidence risk ratios for UKA:TKA by primary variable of interest

Variable of interest	IRR	95% CI	*p*-Value
Race/ethnicity			
Black	0.64	0.58–0.71	< 0.001
Hispanic	0.93	0.84–1.01	0.10
Asian or Pacific Islander	0.97	0.82–1.15	0.72
Native American	0.94	0.68–1.28	0.68
Other	0.99	0.85–1.14	0.84
Socioeconomic status			
Income Q1	0.85	0.79–0.90	< 0.001
Income Q2	0.87	0.82–0.93	< 0.001
Income Q3	0.95	0.90–1.01	0.10
Payer status			
Medicare	0.83	0.79–0.88	< 0.001
Medicaid	0.93	0.84–1.03	0.15
Self-pay	0.93	0.70–1.25	0.65
No charge	1.30	0.54–3.11	0.55
Other	1.07	0.96–1.18	0.20

Reference groups were white (race/ethnicity), income Q4 (socioeconomic status), and private insurance (payer status). CI, confidence interval; IRR, incidence rate ratio

*Covariates* Age was included as a covariate, as was the Elixhauser Comorbidity Index (ECI), calculated using the Elixhauser Stata package that uses 31 patient comorbidities to determine the ECI. The ECI is drawn from a study of the impact of comorbidities on commonly studied outcomes such as length of stay, mortality, and cost [19]. Comorbidities included in the index include hypertension, heart failure, diabetes, anemia, substance use disorders and other psychiatric conditions, cancer, liver disease, and several others. Additionally, we controlled for hospital region (northeast, midwest, south, and west) and sex. Only patients for whom data on all covariates and primary outcome measures were available were included in the analysis.

### Statistical analysis

To assess the differences in rates of UKA:TKA by each primary variable, we used chi-squared tests. We then used multivariable Poisson regression models to examine the effects of each primary variable on incidence rate ratio (IRR) of UKA:TKA, each of which controlled for all other primary variables and covariates. To measure differences in patient characteristics between UKA and TKA groups, we used two-tailed *t*-tests.

Data were analyzed using Stata Statistical Software: Release 18; 2023 (StataCorp; College Station, TX). A *p*-value of < 0.05 was considered statistically significant.

## Results

### Patient population

Table 1 presents the characteristics of our study population. There were 8027 patients who underwent UKA and 606,028 patients who underwent TKA over the study period. A significantly greater percentage of patients undergoing TKA were women (61.5%) compared with patients undergoing UKA (51.2%; *p* < 0.001). Patients who underwent UKA were significantly younger (63.5 ± 10.7 years) than patients who underwent TKA (66.8 ± 9.5 years; *p* < 0.001), and had significantly lower ECI scores (1.8 ± 1.5) than patients who underwent TKA (2.2 ± 1.6; *p* < 0.001).

### Trends in incidence rate of UKA relative to TKA

Utilization of UKA relative to TKA varied significantly by race/ethnicity, income quartile, and payer status (*p* < 0.001 for all). Table 2 presents the full results of outcome modeling by each factor. Compared with White patients, Black patients underwent UKA at a lower relative rate (IRR 0.64, CI 0.58–0.71, *p* < 0.001). Compared with patients in income Q4, patients in income Q1 and Q2 underwent UKA at a lower relative rate (IRR 0.85, CI 0.79–0.90, *p* < 0.001 and IRR 0.87, CI 0.82–0.93, *p* < 0.001; respectively). Compared with patients with private insurance, patients with Medicare underwent UKA at a lower relative rate (IRR 0.83, CI 0.79–0.88, *p* < 0.001).

## Discussion

Using a nationally representative database, we found significant disparities in UKA usage by race, socioeconomic status, and payer status. In line with our hypothesis, Black patients, patients from the lowest income quartiles, and Medicare patients were less likely to undergo UKA compared with white patients, patients from the highest income quartile, and privately insured patients, respectively. These findings persisted despite controlling for many potentially confounding variables, including age, hospital region, and the Elixhauser Comorbidity Index. As TKA is one of the most common procedures in the USA [20], and UKA has emerged as an alternative procedure for isolated unicompartment knee osteoarthritis, it is important to identify any disparities in access to UKA to optimize treatment for a large portion of patients. The presence of differences in UKA utilization demonstrated in this study highlights areas for potential improvement in the delivery of more equitable care.

### Racial disparities in UKA utilization

We found that Black patients underwent UKA at a significantly lower rate than White patients. This is consistent with previous studies, which reported that Black patients had the lowest utilization rate of UKA [16]. Addressing this disparity is important because patients with isolated unicompartmental knee osteoarthritis that meet other indications for and receive UKA have fewer complications, shorter hospital stays, and a faster recovery [4–7]. The causes of this disparity are likely multifaceted. It has been shown that Black patients are less satisfied in communication with their surgeons than White patients; this miscommunication may result in Black patients being less informed about the benefits of UKA over TKA, which may lead to lower utilization rates of UKA [21]. Black patients also have a higher rate of severe osteoarthritis, which may limit the utilization of UKA as an effective treatment [22]. Furthermore, studies have shown that Black patients receive fewer surgical referrals for osteoarthritis and receive referrals to smaller provider networks compared with White patients [23, 24]. This may result in a later presentation to an orthopedic surgeon with more severe osteoarthritis, limiting the utility of UKA. Additionally, in 2019, less than 30% of the orthopedic surgeons who performed TKA also performed UKA [25]. Black patients may have reduced access to the minority of arthroplasty surgeons who perform UKA, thus limiting their likelihood of being offered UKA as an option relative to White patients. Black patients have previously been found to undergo surgery at lower-quality, low-volume hospitals; this historical trend of receiving access to care in lower-quality hospitals may partially be contributing to the disparities observed in our study [26].

### Socioeconomic disparities in UKA utilization

Our findings show that UKA was performed at a significantly lower rate in patients in income Q1 and Q2 compared with patients in income Q4. While past studies have shown this trend for TKA utilization, our study is the first to examine how socioeconomic factors affect UKA utilization [27]. These differences are likely due to risk factors associated with lower socioeconomic status. Previously, it has been shown that lower health literacy has been correlated with lower socioeconomic status [28]. Specifically, in NIS, it has been demonstrated that patients in income Q1 have more social determinants of health disparities (SDHD) than patients in income Q4 [29], which has been linked to lower health literacy [30]. Because of this, patients in lower-income quartiles may be less informed about the differences between UKA and TKA compared with patients in income Q4. As TKA is more common, these patients may choose TKA without understanding the benefits of UKA. Additionally, geographic limitations such as transportation arrangement may limit the access of patients in lower-income quartiles to orthopedic surgeons that perform UKA [4, 31].

Interestingly, studies have shown that UKA has reduced implant costs and decreased utilization of hospital resources, leading to a lower cost for the hospital compared with TKA [10, 32–34]. Peersman et al. reported that choosing UKA resulted in a €2807 cost reduction over TKA [32]. The lower cost of UKA, coupled with comparable outcomes to TKA, may allow for cost-effective expansion of access to arthroplasty care for disadvantaged populations, particularly for patients who may be receiving care at hospitals with fewer resources.

### Payer status disparities in UKA utilization

This study demonstrates that Medicare patients were 17% less likely to receive UKA than privately insured patients. Several factors, including transportation and wait times, may impact a patient’s decision between TKA and UKA. Medicare reimbursement rates are lower than those of private insurers and have decreased since 2000 for orthopedic procedures [35]. Consequently, some hospitals may only accept private insurance, limiting access for patients on Medicare [36]. Furthermore, because surgeons that perform UKA are only a subset of those that perform TKA, finding surgeons that accept Medicare may prove to be challenging for these patients. Lack of accessibility may result in Medicare recipients having to travel substantial distances to receive care with a provider that performs UKA [4, 31]. Additionally, wait times for appointments with these providers are likely longer, creating another barrier to equitable healthcare access [31].

UKA may additionally be a better option for younger patients, as about 8% of TKAs fail within 10 years [37]. Because the conversion of UKA to TKA carries a lower morbidity than revision of a TKA, it may be favored among younger patients as an initial step in treating knee OA. This is supported by our data, which demonstrate the average age of UKA patients to be 63.5 ± 10.7 years, versus 66.8 ± 9.5 years in TKA patients. Since most Medicare beneficiaries are 65 years and older, age may be a confounding factor in our finding that Medicare patients were more likely to undergo TKA. We attempted to minimize this by controlling for age as a covariate in our multivariable regression models to limit the impact of age on the observed effect of payer status on our outcome.

## Limitations

While our study demonstrates the existence of disparities in UKA utilization using a large, nationally representative database, there are limitations. First, as the NIS reports ICD-10 codes for procedures, the accuracy of the data is dependent on appropriate entry of these codes at the point of care. Studies have shown a high degree of accuracy for underlying procedures, for example, 98% for revision TKA, but a lower degree of accuracy around more granular codes such as components used or removal and replacement codes [38]. Consequently, it is possible that patients who underwent UKA may have been misclassified as undergoing TKA, or patients may have not been included altogether. Additionally, as the NIS is a US database, the data collected reflect US classifications of race/ethnicity, socioeconomic status, and insurance, and the patient characteristics and comorbidities reflect those unique to the US arthroplasty patient population; thus, the findings of this study may not be generalizable to other countries. We were also only able to control for covariates present in the database; thus, some comorbidities absent from the Elixhauser Comorbidity Index may have not been accounted for. Radiographic features, patient activity level, and severity of osteoarthritis, important decision-making factors in indications for UKA and TKA, were also not included in the NIS and thus were not accounted for. Additionally, the database does not contain information about patient choice; it may be feasible that patients were offered both TKA and UKA as options but chose TKA for various reasons, including desiring a more definitive treatment that did not carry the risk of requiring a conversion in the future. Similarly, as noted previously, surgeon experience, protocols, and volume may also influence likelihood of undergoing UKA relative to TKA, neither of which can be accounted for in the NIS database. However, if subsets of patients chose or were offered different treatment options on the basis of characteristics inherent to these populations or to the surgeons to whom they had access, this in and of itself represents a disparity and remains important to report and investigate further. Controlling for these factors in an analysis would inevitably mask the existence of disparities, as they are driven by such factors, but initial investigations such as the present study that primarily identify disparities remain important for establishing where they exist and highlighting directions for future research into underlying drivers. Furthermore, because the NIS is an inpatient database, we did not capture cases that were done on an outpatient basis. As knee arthroplasty has begun moving toward an outpatient setting, this may have biased our cohort [39]. Other databases that include outpatient data, such as National Surgical Quality Improvement Program (NSQIP), were considered but did not include key metrics that we wanted to test, such as payer or socioeconomic status. Because of this, our results should be interpreted in the context of our cohort being limited to inpatient cases. This is especially important when considering our findings on payer status, as Medicare removed TKA from the inpatient-only list in 2018. Prior to this, Medicare-funded TKAs were only performed in an inpatient setting, while privately funded TKAs were performed in both inpatient and outpatient settings. Therefore, our results may have not captured all TKAs performed in privately insured patients, which may have affected our conclusions about the association between payer status and likelihood of receiving UKA:TKA. However, it has previously been shown that the percentage of privately insured TKAs that occurred in the outpatient setting prior to 2018 was small, and only greatly increased after the removal of TKA from the Medicare inpatient-only list, involving only half our study period [40]. Finally, because our study is observational, our findings can only imply a correlation between race, socioeconomic status, or payer status and UKA utilization, not causation.

## Conclusions

In summary, we examined disparities in the utilization of UKA compared with TKA using a national database. We demonstrated that Black patients were less likely to undergo UKA than White patients. Furthermore, lower-income-quartile and Medicare patients were less likely to receive UKA compared with highest-income-quartile and privately insured patients. Further research may help determine specific causes and solutions for these disparities.

## Supplementary Information

Additional file 1.

Additional file 2.

## Data Availability

The anonymized data collected in the National Inpatient Sample are publicly available for purchase online via the Healthcare Cost and Utilization Project (HCUP): https://hcup-us.ahrq.gov/tech_assist/centdist.jsp
