# Supplementary material for: Racial, socioeconomic, and payer status disparities in utilization of unicompartmental knee arthroplasty in the USA
Source: Knee Surg Relat Res. 2025 Jan 9;37:2. doi: 10.1186/s43019-024-00227-4 (PMC11720326; doi:10.1186/s43019-024-00227-4)
Supplement: Supplementary file 1 — Additional file 1. [file 43019_2024_227_MOESM1_ESM.docx]

**Supplemental Table 1.** ICD-10 codes utilized to isolate UKA and TKA patients.

| **TKA** | | | **UKA** |
| --- | --- | --- | --- |
| ICD-10-P-0SRC069 | ICD-10-P-0SRD0J9 | ICD-10-P-0SRU0KZ | ICD-10-P-0SRC0L9 |
| ICD-10-P-0SRC06A | ICD-10-P-0SRD0JA | ICD-10-P-0SRU07Z | ICD-10-P-0SRC0LA |
| ICD-10-P-0SRC06A | ICD-10-P-0SRD0JZ | ICD-10-P-0SRV0J9 | ICD-10-P-0SRC0LZ |
| ICD-10-P-0SRC07Z | ICD-10-P-0SRD0KZ | ICD-10-P-0SRV0JA | ICD-10-P-0SRD0L9 |
| ICD-10-P-0SRC0J9 | ICD-10-P-0SRT0J9 | ICD-10-P-0SRV0JZ | ICD-10-P-0SRD0LA |
| ICD-10-P-0SRC0JA | ICD-10-P-0SRT0JA | ICD-10-P-0SRV0KZ | ICD-10-P-0SRD0LZ |
| ICD-10-P-0SRC0JZ | ICD-10-P-0SRT0JZ | ICD-10-P-0SRV07Z |  |
| ICD-10-P-0SRC0KZ | ICD-10-P-0SRT07Z | ICD-10-P-0SRW0J9 |  |
| ICD-10-P-0SRD069 | ICD-10-P-0SRT0KZ | ICD-10-P-0SRW0JA |  |
| ICD-10-P-0SRD06A | ICD-10-P-0SRU0J9 | ICD-10-P-0SRW0JZ |  |
| ICD-10-P-0SRD06Z | ICD-10-P-0SRU0JA | ICD-10-P-0SRW0KZ |  |
| ICD-10-P-0SRD07Z | ICD-10-P-0SRU0JZ | ICD-10-P-0SRW07Z |  |
