# Supplementary material for: Racial, socioeconomic, and payer status disparities in utilization of unicompartmental knee arthroplasty in the USA
Source: Knee Surg Relat Res. 2025 Jan 9;37:2. doi: 10.1186/s43019-024-00227-4 (PMC11720326; doi:10.1186/s43019-024-00227-4)
Supplement: Supplementary file 2 — Additional file 2. [file 43019_2024_227_MOESM2_ESM.docx]

**Supplemental Table 2.** Quartile Ranges of Median Household Income by Patient Zip Code by Year

| Year | Q1 | Q2 | Q3 | Q4 |
| --- | --- | --- | --- | --- |
| 2016 | 1 – 42,999 | 43,000 - 53,999 | 54,000 - 70,999 | 71,000+ |
| 2017 | 1 - 43,999 | 44,000 - 55,999 | 56,000 - 73,999 | 74,000+ |
| 2018 | 1 - 45,999 | 46,000 - 58,999 | 59,000 - 78,999 | 79,000+ |
| 2019 | 1 - 47,999 | 48,000 - 60,999 | 61,000 - 81,999 | 82,000+ |
| 2020 | 1 - 49,999 | 50,000 - 64,999 | 65,000 - 85,999 | 86,000+ |

Definition of quartile ranges taken from NIS description of data elements
